# Using HIV-attributable mortality to assess the impact of antiretroviral therapy on adult mortality in rural Tanzania

**DOI:** 10.3402/gha.v7.21865

**Published:** 2014-03-20

**Authors:** Chifundo Kanjala, Denna Michael, Jim Todd, Emma Slaymaker, Clara Calvert, Raphael Isingo, Alison Wringe, Basia Zaba, Mark Urassa

**Affiliations:** 1 National Institute for Medical Research, Mwanza, Tanzania; 2 London School of Hygiene and Tropical Medicine, London, UK

**Keywords:** HIV-attributable mortality, ART, HDSS, InterVA model, serological survey, verbal autopsy

## Abstract

**Background:**

The Tanzanian national HIV care and treatment programme has provided free antiretroviral therapy (ART) to HIV-positive persons since 2004. ART has been available to participants of the Kisesa open cohort study since 2005, but data to 2007 showed a slow uptake of ART and a modest impact on mortality. Additional data from the 2010 HIV serological survey provide an opportunity to update the estimated impact of ART in this setting.

**Methods:**

The Kisesa Health and Demographic Surveillance Site (HDSS) has collected HIV serological data and demographic data, including verbal autopsy (VA) interviews since 1994. Serological data to the end of 2010 were used to make two estimates of HIV-attributable mortality, the first among HIV positives using the difference in mortality between HIV positives and HIV negatives, and the second in the population using the difference between the observed mortality rate in the whole population and the mortality rate among the HIV negatives. Four time periods (1994–1999, 2000–2004, 2005–2007, and 2008–2010) were used and HIV-attributable mortality estimates were analysed in detail for trends over time. A computer algorithm, InterVA-4, was applied to VA data to estimate the HIV-attributable mortality for the population, and this was compared to the estimates from the serological survey data.

**Results:**

Among HIV-positive adults aged 45–59 years, high mortality rates were observed across all time periods in both males and females. In HIV-positive men, the HIV-attributable mortality was 91.6% (95% confidence interval (CI): 84.6%–95.3%) in 2000–2004 and 86.3% (95% CI: 71.1%–93.3%) in 2008–2010, while among women, the HIV-attributable mortality was 87.8% (95% CI: 71.1%–94.3%) in 2000–2004 and 85.8% (95% CI: 59.6%–94.4%) in 2008–2010. In the whole population, using the serological data, the HIV-attributable mortality among men aged 30–44 years decreased from 57.2% (95% CI: 46.9%–65.3%) in 2000–2004 to 36.5% (95% CI: 18.8%–50.1%) in 2008–2010, while among women the corresponding decrease was from 57.3% (95% CI: 49.7%–63.6%) to 38.7% (95% CI: 27.4%–48.2%). The HIV-attributable mortality in the population using estimates from the InterVA model was lower than that from HIV sero-status data in the period prior to ART, but slightly higher once ART became available.

**Discussion:**

In the Kisesa HDSS, ART availability corresponds with a decline in adult *overall mortality*, although not as large as expected. Using InterVA to estimate HIV-attributable mortality showed smaller changes in HIV-related mortality following ART availability than the serological results.

Since the first AIDS case was identified in the 1980s, HIV/AIDS has been a major public health challenge, particularly in sub-Saharan Africa, where the burden of the disease is greatest. Prior to the introduction of free antiretroviral therapy (ART) treatment, the age-standardised mortality rates among HIV-positive adults were high, being of the order of 9–25 times those of the uninfected (1). In some populations, the probability of dying between 15 and 60 years of age reached 60% among men (2). Results from modelling vital registration data and antenatal clinic surveillance data in 2000 showed that 40% of deaths in South African adults aged 15–49 years were HIV related (3).

The positive effect of ART use on mortality (4–6), and support from donor organisations, encouraged the introduction of free national ART programmes in many sub-Saharan African countries from 2004. Data from longitudinal demographic surveillance in several countries have confirmed the impact of ART on HIV mortality in poor-resource settings. In rural, northern Malawi there was a 19% decline in AIDS-related mortality amongst adults aged 15–59 within 8 months of ART becoming available (7), and a 68% decline over the 5 years from 2004–2009 (8). In northern KwaZulu-Natal, South Africa, HIV-related mortality in adults aged 25–49 years was reduced by 22% in women and 29% in men between 2002–2003 and 2004–2006 following the introduction of ART in 2004 (9). In South-west Uganda, there was a 19% decline in the HIV population-attributable fraction of mortality among adults aged 15–59 in the 5-year period after ART introduction in 2004 (10).

This study aims to assess the long-term impact of ART on HIV/AIDS mortality in Kisesa, Tanzania. In this observational study, care and treatment services were provided by local health facilities in line with national policies. While residents of Kisesa had initial access to ART in 2005, it was only available at a referral hospital 20 km away. Referrals from the local voluntary counselling and testing (VCT) to the regional hospitals in Mwanza were not well organised. In addition, travel and other logistics were difficult for the Kisesa residents. By 2007, only 3% of adults in need of treatment were receiving ART (11) but even at this low level of coverage there was evidence to suggest ART was having an impact on adult mortality (12). To improve the situation, the Kisesa study group (TAZAMA study) facilitated referrals to the hospital to improve uptake of ART in the study area during this ART introduction period. However from 2008, ART became available at a local clinic in Kisesa which allowed easier access and improved uptake of ART, with a corresponding increase in the proportion of adults in need receiving treatment (unpublished data). In this study, data from the serological surveys and demographic follow-up until 2010 are used to examine trends in HIV-attributable mortality, both among HIV-positive adults and in the whole population, over time.

## Methods

The Kisesa open cohort study consists of six villages in Mwanza region in Tanzania. In 2010, the total population was approximately 30,000. In the period 1994–1998, life expectancy was 43–44 years, and approximately 50% of the population were younger than age 15 (13). Biannual health demographic surveillance system (HDSS) rounds since 1994 have measured births, deaths, and migration in the resident population, with mortality among adults aged 15–59 years estimated at 39% in 1994, and 22% in 2009 (14). Annual adult external migration (out of the Kisesa cohort) has been shown to be 10% in males and 12% in females in 1994–1998 (15), and around 8% among both male and female adults in 2012 (16) (unpublished thesis). Verbal autopsies (VAs) have been conducted to track the leading causes of death (13), in which the main care giver or the most reliable informant available is interviewed to capture the signs and symptoms that the deceased had in the time leading to their death (17). The current VA questionnaire is taken from the recommended WHO tool (18), although previous versions were very similar, and included the same questions on signs and symptoms required for the ascertainment of cause of death.

Since 1994, six serological surveys have been carried out to measure HIV prevalence and incidence in the cohort population; the first was restricted to adults aged between 15 and 50 years of age while subsequent serological surveys were conducted amongst all adults aged 15 years and above (19, 20). The serological surveys were done at 2- to 3-year intervals and the first four were done in 1994/1995, 1996/1997, 1999/2000, and 2003/2004 (20). The other two were done in 2006/2007 and in 2010. They included questionnaire-based interviews and thereafter, consenting adults provided a finger prick blood sample, which was tested for HIV at the National Institute for Medical Research laboratory in Mwanza (12). Almost all of the respondents aged 15+ who got interviewed also got tested for HIV. In the first four serological surveys, of the 28,591 interviews done, 28,523 interviewees got tested (20). Attendance rates were between 61 and 86% across the sexes and surveys. Non-attendance is mainly due to absence from home during the survey and not rejecting to get tested for HIV (20).

HIV prevalence in Kisesa HDSS increased steadily from about 6% in 1994–1995 until its peak at about 8.3% in 2000–2001 (20). It then went down to 7.6% in 2004 and then 7.1% in 2007 (21). By 2011, the prevalence was 6% (22).

### Statistical methods

For this analysis, adult person-years of residence were assessed from entry into the HDSS either at age 15 years, through in-migration, or since the start of the HDSS in 1994, until their exit due to out-migration, death or censoring at the time of the latest HDSS survey. Returning migrants (the individuals re-entering the study following out-migration) contribute person-years only for the time they were actually resident in the study site. Person-years were stratified by sex and further by age group, and years related to ART rollout. Age was stratified into three age groups (15–29, 30–44 and 45–59 years, respectively). Person-years of exposure were also stratified into different HIV status categories: HIV negative, HIV positive and HIV status unknown. HIV-negative person-years were obtained by summing the time between successive HIV-negative tests, up to 5 years following the last recorded HIV-negative test, and the half the period between a negative test and a subsequent positive test in which the sero-conversion interval is less than 5 years. HIV-positive person-years were obtained from the time following an HIV-positive test. HIV unknown person-years were obtained from summing the time observed for those never tested or not yet tested (i.e. before first test, whether or not that first test was positive or negative) and the period more than 5 years following the last negative test. Mortality rates were obtained by dividing the number of deaths by the person-years at risk, and 95% confidence intervals (95% CI) obtained from the normal approximation to the log rate (23).

Analysis was carried out separately based on the availability of ART, with four time periods identified as follows: 1) More than 5 years before ART (i.e. no treatment available) from 1994 to 2000; 2) 5 years leading to the introduction of ART (2000–2004); 3) a 3-year roll-out phase immediately following the introduction of ART when ART was assumed to be only partially available (2005–2007); and 4) a phase when ART was widely and locally available (2008–2010).

Mortality rates, *M*_*x*_, were calculated by dividing the deaths, *D*_*x*_, by the person-years of residence, *L*_*x*_, for each age and sex group (designated as *x* in the equation), $M_{x}=\frac{D_{x}}{L_{x}}$ and are expressed as mortality per 1,000 person-years (1,000 pyrs).

### HIV-attributable fraction among HIV-positive adults

Within each period, mortality rates were calculated separately among the HIV positives ($M_{x}^{HIV+}$) and HIV negatives ($M_{x}^{HIV-}$). The HIV-attributable mortality among the positives was calculated as the excess mortality among the HIV positives after subtracting the expected non-AIDS mortality among the HIV positives, as given by:

$$M_{x}^{HIV+attrib}=\frac{D_{x}^{HIV+attrib}}{L_{x}^{HIV+}}=M_{x}^{HIV+}-M_{x}^{HIV-},$$

where $M_{x}^{HIV+attrib}$ is the age-specific mortality due to HIV among the HIV positive, $D_{x}^{HIV+attrib}$ are the deaths due to HIV among the HIV positive, $L_{x}^{HIV+}$ are the person-years lived by the HIV positives, $M_{x}^{HIV+}$ is the overall mortality among the HIV positives and $M_{x}^{HIV-}$ is the mortality among the HIV negatives. HIV-attributable fractions among the HIV positives were then calculated by dividing $M_{x}^{HIV+attrib}$ by $M_{x}^{HIV+}$.

### HIV-attributable fraction among the whole population

The HIV-attributable mortality in the population was calculated as:

$$M_{x}^{PopAttrib}=M_{x}^{TotalPop}-M_{x}^{HIV-},$$

where $M_{x}^{PopAttrib}$ is the age-specific mortality rate due to HIV in the population and $M_{x}^{TotalPop}$ is the mortality rate in the total population. Similarly, HIV-attributable mortality fractions in the entire population were calculated by dividing $M_{x}^{PopAttrib}$ by $M_{x}^{TotalPop}$.

### The InterVA4 algorithm

The InterVA4 algorithm is a probability-based model for assigning causes of death on the basis of verbal autopsy (VA) interview reports from the main caregivers of the deceased (24). In this study, it was used to assign the cause of deaths occurring in the study population for the period 1994 to 2010. The InterVA4 model takes responses from a VA interview and uses these inputs to determine probabilistic causes of deaths.

InterVA4 is not intended to diagnose the cause of death for an individual case, but the probabilities of any cause of death can be added up across the population to give the expected total number of deaths for that cause. In this study, the InterVA algorithm was applied to all deaths where a VA had been performed in the Kisesa cohort in order to estimate the probability of HIV being the cause of the death. These probabilities were added up over all deaths to obtain the number of HIV-related deaths and the HIV-attributable mortality in the population. The population HIV-attributable mortality fraction from the InterVA4 model was compared to the HIV-attributable mortality fraction from the serological data in Kisesa.

## Results

### Characteristics of data

Table 1 shows the crude mortality rates among adults aged 15 years and over by time period. Overall crude mortality in the adult population has gone down from 13 deaths per 1,000 person-years in the period at least 5 years before ART, to 12 deaths per 1,000 person-years in the 5-year period leading to the introduction of ART, to eight deaths per 1,000 person-years during the period of widespread availability of ART. There was strong evidence for a decline in mortality rates between the period 5 years before ART introduction and the period when ART became widely available (HR: 0.61, 95% CI: 0.54–0.69, *p*<0.000).

**Table 1:** Number of subjects, person-years and deaths by ART phase in adults aged 15 years and above in the HDSS population

ART phase	Number of subjects	Person-years	Deaths	Mortality rate per 1,000 pyrs	95% CI
5+ years before ART (1994–2000)	23,316	60,057	783	13.0	12.0–14.0
0–5 years before ART (2000–2004)	26,031	63,248	780	12.3	11.5–13.1
During ART introduction (2005–2007)	22,253	48,309	523	10.8	9.8–11.8
After ART became available (2008–2010)	22,387	46,274	370	8.0	7.2–8.8

ART=antiretroviral therapy; CI=confidence interval; HDSS=Health and Demographic Surveillance Site; pyrs=person-years.

### Age-specific mortality rates by HIV status and ART availability

Tables 2 and 3, show person-years, deaths and mortality rates stratified by age, HIV status and ART availability for males and females, respectively. Mortality among the HIV negatives was generally lower than among HIV positives for both sexes and all age groups across the four time periods considered. The levels of mortality for those with unknown HIV status were generally in between those of the HIV negatives and those of the HIV positives. The mortality rates among the HIV positives declined over time following the introduction of ART, while the HIV-negative mortality rates of both males and females fluctuated over time without a clearly defined trend. In each time period and HIV status group, mortality increased by age, except in the period 5 or more years before introduction of ART during which it was highest in the 30–44 years age group for HIV-positive males and in the period of wide ART availability where for females it was highest in the 15–29 age group. The decline in mortality is clearer and more consistent in the HIV-positive women compared to men. However, the CI are overlapping and wide indicating high uncertainty surrounding the magnitude of the decline in the mortality reported.

**Table 2:** Age-specific person-years, deaths and mortality rates per 1000 person-years by HIV status and ART availability observed in Kisesa open cohort among male adults aged 15–59 in the period 1994–2010

	HIV negative			HIV positive			HIV status unknown		
			
Age interval	Person-years	Deaths	Mortality rate (95% CI)	Person-years	Deaths	Mortality rate (95% CI)	Person-years	Deaths	Mortality rate (95% CI)
5+ years before ART (1994–1999)									
15–29	7618.5	21	2.8 (1.8–4.2)	170.5	7	41.1 (19.6–86.1)	7081.8	38	5.4 (3.9–7.4)
30–44	4015.3	26	6.5 (4.4–9.5)	260.3	34	130.6 (93.3–182.8)	3848.8	57	14.8 (11.4–19.2)
45–59	530.3	8	15.1 (7.5–30.2)	44.6	5	112.0 (46.6–269.1)	3495.6	69	19.7 (15.6–25.0)
0–5 years before ART (2000–2004)									
15–29	7382.8	9	1.2 (0.6–2.3)	252.9	8	31.6 (15.8–63.3)	7816.7	44	5.6 (4.2–7.6)
30–44	4335.5	24	5.5 (3.7–8.3)	354.7	28	78.9 (54.5–114.3)	4084.3	61	14.9 (11.6–19.2)
45–59	2115.0	29	13.7 (9.5–19.7)	133.1	22	165.2 (108.8–251.0)	1843.5	27	14.6 (10.0–21.4)
ART introduction (2005–2007)									
15–29	5723.7	16	2.8 (1.8–4.6)	145.1	6	41.3 (18.6–92.0)	5681.6	20	3.5 (2.3–5.5)
30–44	3061.3	27	8.8 (6.0–12.9)	277.5	21	75.7 (49.3–116.1)	3417.5	29	8.5 (5.9–12.2)
45–59	1789.3	27	15.1 (10.3–22)	112.0	10	89.3 (48.0–166.0)	1457.0	23	15.8 (10.5–23.8)
ART available (2008–2010)									
15–29	4558.6	15	3.3 (2.0–5.5)	85.0	3	35.3 (11.4–109.5)	6182.4	24	3.9 (2.6–5.8)
30–44	2075.4	4	1.9 (0.7–5.1)	181.3	8	44.1 (22.1–88.3)	4303.0	23	5.3 (3.6–8.0)
45–59	1420.1	8	5.6 (2.8–11.3)	141.7	9	63.5 (33.1–122.1)	1679.6	24	14.3 (9.6–21.3)

ART=antiretroviral therapy; CI=confidence interval.

**Table 3:** Age-specific person-years, deaths and mortality rates per 1000 person-years by HIV status and ART availability observed in Kisesa open cohort among female adults aged 15–59 in the period 1994–2010

	HIV negative			HIV positive			HIV status unknown		
			
Age interval	Person-years	Deaths	Mortality rate (95% CI)	Person-years	Deaths	Mortality rate (95% CI)	Person-years	Deaths	Mortality rate (95% CI)
5+ years before ART (1994–1999)									
15–29	6671.8	15	2.3 (1.4–3.7)	408.5	21	51.4 (33.5–78.8)	7787.0	46	5.9 (4.4–7.9)
30–44	5153.5	19	3.7 (2.4–5.8)	305.7	31	101.4 (71.3–144.2)	3081.6	45	14.6 (10.9–19.6)
45–59	632.6	3	4.7 (1.5–14.7)	18.5	3	161.9 (52.2–501.9)	3503.2	51	14.6 (11.1–19.2)
0–5 years before ART (2000–2004)									
15–29	6536.0	19	2.9 (1.9–4.6)	407.9	17	41.7 (25.9–67.0)	8418.8	43	5.1 (3.8–6.9)
30–44	6166.7	34	5.5 (3.9–7.7)	482.6	39	80.8 (59.0–110.6)	2766.0	42	15.2 (11.2–20.5)
45–59	3139.6	35	11.1 (8.0–15.5)	106.1	10	94.3 (50.7–175.2)	1251.6	17	13.6 (8.4–21.8)
ART introduction (2005–2007)									
15–29	4958.0	12	2.4 (1.4–4.3)	217.8	11	50.5 (28.0–91.2)	6034.2	22	3.6 (2.4–5.5)
30–44	4699.5	19	4.0 (2.6–6.3)	429.9	18	41.9 (26.4–66.5)	2212.6	19	8.6 (5.5–13.5)
45–59	2619.0	20	7.6 (4.9–11.8)	116.7	10	85.7 (46.1–159.3)	948.4	10	10.5 (5.7–19.6)
ART available (2008–2010)									
15–29	4091.1	6	1.5 (0.7–3.3)	155.0	5	32.3 (13.4–77.5)	6946.9	17	2.4 (1.5–3.9)
30–44	3422.1	9	2.6 (1.4–5.1)	441.5	10	22.6 (12.2–42.1)	2977.2	19	6.4 (4.1–10.0)
45–59	2261.4	7	3.1 (1.5–6.5)	144.7	3	20.7 (6.7–64.3)	1216.7	16	13.1 (8.1–21.5)

ART=antiretroviral therapy; CI=confidence interval.

### HIV-attributable mortality in the Kisesa HDSS

HIV-attributable mortality estimates are presented in Tables 4 and 5 for males and females, respectively. In HIV-positive people of both sexes, and across all of the periods, around 90% of the deaths among those aged 15–59 years of age are due to HIV. There was little change in this proportion in the periods after the introduction of ART, suggesting that mortality attributable to HIV among HIV-positive people is still very high. The HIV-attributable mortality in the entire population appears to decline over time. In the periods before ART, more than 40% of the deaths in both males and females aged 15–44 years of age were attributable to HIV. After the introduction of ART, the proportion of mortality attributable to HIV in the 15–44 year age groups reduced to less than 40% among the males, although among females the pattern was less clear.

**Table 4:** HIV-attributable mortality for males aged 15–59 in Kisesa open cohort from serological data and InterVA model, 1994–2010

	Using sero-status data			Using InterVA4		
		
Age interval	*Deaths	HIV-attributable mortality fractions among the HIV- positive deaths (95% CI)	HIV-attributable mortality fractions in the entire population (95% CI)	**Deaths	HIV-deaths assigned by InterVA4	% deaths attributable to HIV using InterVA4
5+ years before ART (1994–1999)						
15–29	66	94.1 (83.5–97.6)	41.1 (28.1–51.6)	64	19	29.7
30–44	117	95.3 (91.9–97.3)	56.2 (46.2–64.3)	109	39	35.8
45–59	82	85.0 (30.2–96.2)	27.6 (−19.7–55.5)	71	12	16.9
0–5 years before ART (2000–2004)						
15–29	61	96.1 (88.5–98.7)	68.5 (53.5–78.4)	36	8	22.2
30–44	113	93.0 (87.5–96.1)	57.2 (46.9–65.3)	52	11	21.2
45–59	78	91.6 (84.6–95.3)	26.2 (15.4–35.6)	44	7	15.9
ART introduction (2005–2007)						
15–29	42	91.5 (50.4–98.0)	11.8 (−12.5–30.2)	34	6	17.6
30–44	77	79.8 (41.5–92.3)	12.7 (−4.9–27.1)	64	14	21.9
45–59	60	87.9 (61.1–95.7)	13.5 (−3.0–27.0)	56	11	19.6
ART available (2008–2010)						
15–29	42	91.4 (74.6–96.6)	14.2 (1.2–25.4)	27	8	29.6
30–44	35	93.2 (86.5–96.6)	36.5 (18.8–50.1)	29	16	55.2
45–59	41	86.3 (71.1–93.3)	35.8 (22.7–46.5)	29	5	17.2

* Of the *814 male deaths among adults aged 15–59

** 615 (76%) had VAs thus the difference in the number of deaths with a sero-status and those with a corresponding VA. ART=antiretroviral therapy; CI=confidence interval; VAs=verbal autopsies.

**Table 5:** HIV-attributable mortality for females aged 15–59 in Kisesa open cohort from serological data and InterVA model, 1994–2010

	Using sero-status data			Using InterVA4		
		
Age interval	*Deaths	HIV-attributable mortality fractions among the HIV- positive deaths (95% CI)	HIV-attributable mortality fractions in the entire population (95% CI)	**Deaths	HIV deaths assigned by InterVA4	% deaths attributable to HIV using InterVA4
5+ year before ART (1994–1999)						
15–29	82	95.4 (90.5–97.8)	58.2 (44.6–68.3)	78	39	50.0
30–44	95	96.2 (93.0–98.0)	65.3 (55.9–72.7)	83	43	51.8
45–59	57	97.0 (77.4–99.6)	61.8 (14.3–82.0)	57	10	17.5
0–5 year before ART (2000–2004)						
15–29	79	93.2 (86.3–96.6)	44.9 (30.7–56.1)	51	16	31.4
30–44	115	93.5 (89.3–96.1)	57.3 (49.7–63.6)	61	30	49.2
45–59	62	87.8 (71.1–94.3)	19.1 (12.0–25.5)	35	4	11.4
ART introduction (2005–2007)						
15–29	45	92.0 (50.6–98.3)	32.4 (2.8–52.1)	41	13	31.7
30–44	56	93.2 (80.0–97.7)	57.9 (42.0–69.2)	50	14	28.0
45–59	40	93.9 (81.4–97.8)	37.3 (24.0–48.0)	36	8	22.2
ART available (2008–2010)						
15–29	28	96.5 (91.4–98.6)	44.9 (25.2–59.1)	19	8	42.1
30–44	38	85.8 (72.8–92.6)	38.7 (27.4–48.2)	28	15	53.6
45–59	26	85.8 (59.6–94.4)	39.5 (28.1–48.9)	17	5	29.4

* Of the *723 female deaths among adults aged 15–59

** 556 (77%) had VAs thus the difference in the number of deaths with a sero-status and those with a corresponding VA. ART=antiretroviral therapy; CI=confidence interval; VAs=verbal autopsies.

### Evaluating InterVA4 based HIV/AIDS mortality

Table 4 (males) and 5 (females) show the population HIV-attributable mortality from the InterVA4 model compared to the population HIV-attributable mortality from the serological data.

The InterVA-4 model gave higher HIV-attributable mortality among the females compared to the males. The exception is the period of ART introduction in which the proportions of deaths attributable to HIV are higher among the males than the females in all age groups. Comparison of the InterVA-4 estimates to those obtained from serological data show that for the pre-ART period, InterVA-4 provides lower estimates of mortality attributable to HIV in the population. However, in the period of ART introduction and the subsequent period of ART availability, the InterVA model tended to overestimate the population-level HIV-attributable mortality in both males and females.

## Discussion

This paper uses age-specific mortality rates and HIV-attributable mortality fractions to assess the impact of ART availability on adult mortality in the Kisesa HDSS. The results show that the availability of ART to HIV-positive participants has so far yielded a slow decline in adult mortality, six years after it was introduced in 2005. However, the coverage of ART in Kisesa was very low, estimated at 3% for the first 3 years after it was introduced (11, 12). The availability of ART has improved over time, but the impact on population-level mortality may still take some time to observe. Although the methods used in this paper (population attributable fractions among the HIV positives and in the entire population) are different from the exponential regression and hazard rate ratios used previously in this setting (12), the results from these two studies broadly agree in showing that the impact of ART is modest, even with the additional data from the most recently completed serological survey in 2010.

This decline in mortality in Kisesa is less than that reported in other sub-Saharan countries where ART has been introduced, including sites collecting similar community-level serological and demographic data in Uganda (10), South Africa (9) and Malawi (8, 25). Results from the Karonga Prevention Study (KPS), which is a similar mixture of semi-urban and rural settings as the Kisesa open cohort, showed a mortality decline of 21% (95% CI: 2–36%) after ART introduction (between 2004–05 and 2008–09), especially when ART became available within the KPS area (8). These differences are likely to be partially explained by different levels of uptake of HIV testing services. For example, in Kisesa, VCT uptake among HIV-positive people during the serological surveys has been persistently low: 14% in 2003–2004 and approximately 25% in 2006–2007 (26). Much higher levels of knowledge of HIV status have been reported in Malawi (95%) in 2007–2008 where opt-out testing and door-to-door delivery of test results has been offered during serological surveys. Similarly, in Masaka in Uganda, where HIV testing is offered at the household during the annual survey rounds, 56% of HIV-positive adults knew their status in 2008 (11). It is most likely that the impact of ART on mortality will be realised only when wider coverage of VCT and ART are achieved in Kisesa.

Measuring HIV-attributable mortality is important for the evaluation of ART delivery programmes (27). However, it can only be properly measured with good serological data from a representative population-based cohort, and there are few such cohorts in most countries affected by the HIV epidemic. Studies on HIV-positive patients attending clinics can provide estimates of the impact of ART on mortality (28), but do not capture the impact of those who do not know their HIV status, or who have not been assessed for eligibility for ART. Other studies have shown that monitoring burial grounds can estimate the impact of ART on HIV-related mortality, although this does depend on the local culture surrounding death, and the proportion of people using public cemeteries (29).

We compared the population HIV-attributable fractions directly calculated from the sero-status of those who died, and the HIV-attributable mortality from VA where the cause of death had been assigned using the InterVA-4 algorithm. The comparison showed that in the pre-ART period, InterVA tended to provide lower estimates of the contribution of HIV to mortality, but in the period when ART was available, InterVA overestimated the contribution of HIV to mortality. Two other studies have used VA data, one in Kenya and the other one in Ethiopia, comparing the InterVA assessment with the causes of death assigned by physicians (30, 31). In the Kenyan study, HIV-attributable mortality was underestimated by InterVA compared to the physician assignment, but gave similar HIV-attributable mortality fractions when tuberculosis and HIV/AIDS were combined (30). The Ethiopian study on the other hand compared HIV-attributable deaths as assigned by InterVA and those from known hospital diagnosis and HIV status of the deceased (31). The results from this study seem to also suggest that InterVA performs better when HIV and TB are combined. In Kisesa, combining TB and HIV/AIDS-related deaths from InterVA-4 did not alter the results substantially as a relatively small proportion of deaths were assigned to TB (data not shown). On the other hand, a study in Agincourt, South Africa suggests broadly similar HIV-attributable mortality trends over time between InterVA assessment and physician assignment of cause of death (32). Further work may be needed to confirm the usefulness of the InterVA algorithm in assessing HIV-attributable mortality in sub-Saharan Africa.

There are some limitations related to this study. The numbers of deaths used in the calculation of age-specific HIV-attributable mortality fractions are quite small, so the results may be due to random variation and should therefore be interpreted with caution. We did not adjust for potential confounders such as socio-economic status, which may affect the relationship between availability of ART and mortality. We also did not take into consideration the selection effect that migration may bring since those who migrate out are not followed-up to observe their mortality. However, we have no significant reasons to think that those who migrate both in and out of the study area would exhibit different mortality patterns to those of their non-moving counterparts. Participants who tested HIV negative and either had no subsequent test, or tested positive in the subsequent test done more than 5 years after the negative test, were assumed to be HIV negative for up to 5 years. They were then classified as unknown until the date of testing positive. This assumption was made after observing that 95% of those testing HIV negative in a particular round of serological survey remained negative in the ensuing 5 years. We also did sensitivity analysis (data not shown) assuming 2 and 10 years cut-offs for changing classifications of the HIV negative into the unknown group. We did not observe any significant changes in the mortality, therefore we think that the cut-off at 5 years in not unreasonable.

In conclusion, the current study has confirmed a decline in HIV-attributable mortality among HIV positives and all adults in the study population. The decline is not as pronounced as has been experienced in similar study populations in Uganda, Malawi and South Africa, even after 3 years of availability of ART at a clinic in Kisesa, suggesting that greater efforts are needed to improve VCT uptake and treatment initiation for those needing ART in this setting. Analysis of the retention rates for those on ART should be considered in order to confirm the duration of time that individuals starting ART are remaining on treatment.
